# Chemical profiling of *Curatella americana* Linn leaves by UPLC-HRMS and its wound healing activity in mice

**DOI:** 10.1371/journal.pone.0225514

**Published:** 2020-01-13

**Authors:** Mayara Amoras Teles Fujishima, Dayse Maria Cunha Sá, Carolina Miranda de Sousa Lima, José Adolfo H. M. Bittencourt, Washington Luiz Assunção Pereira, Abraão de Jesus Barbosa Muribeca, Consuelo Yumiko Yoshioka e Silva, Milton Nascimento da Silva, Francisco Fábio Oliveira de Sousa, Cleydson B. R. dos Santos, Jocivania Oliveira da Silva

**Affiliations:** 1 Postgraduate Program of Pharmaceutical Innovation, Federal University of Amapá, Macapá, AP, Brazil; 2 Laboratory of Toxicology, Department of Biological and Health Sciences, Federal University of Amapá, Macapá, AP, Brazil; 3 Laboratory of Modeling and Computational Chemistry, Department of Biological and Health Sciences, Federal University of Amapá, Macapá, AP, Brazil; 4 Laboratory of Animal Pathology, Institute of Animal Production, Federal Rural University of Amazônia, Belém, Brazil; 5 Laboratory of Liquid Chromatography, Department of Chemistry, Federal University of Pará, Belém, Brazil; Tallinn University of Technology, ESTONIA

## Abstract

Based on ethnopharmacological studies, a lot of plants, as well as its compounds, have been investigated for the potential use as wound healing agents. In Brazil, *Curatella americana* is traditionally used by local people to treat wounds, ulcers and inflammations. However, to the best of our knowledge, its traditional use in the treatment of wounds has not been validated by a scientific study. Here, some compounds, many of them flavonoids, were identified in the hydroethanolic extract from the leaves of *C*. *americana* (HECA) by LC-HRMS and LC-MS/MS. Besides that, solutions containing different concentrations of HECA and a gel produced with this extract were evaluated for its antimicrobial, coagulant and wound healing activities on an excision mouse wound model as well as its acute dermal safety. A total of thirteen compounds were identified in HECA, mainly quercetin, kaempferol and glucoside derivatives of both, besides catechin and epicatechin known as wound healing agents. The group treated with 1% of HECA exhibited highest wound healing activity and best rate of wound contraction confirmed by histopathology results. The present study provides scientific evidence of, this extract (HECA) possess remarkable wound healing activity, thereby, supporting the traditional use.

## Introduction

Many variables influence wound healing, such as age, nutritional status, infections and, especially, the simultaneous occurrence of diabetes and coagulation problems, generating a higher incidence and prevalence of chronic wounds. Non-healing wounds remain a public health problem resulting in social disorders in patients requiring increasingly effective treatment and consequent high financial costs [1,2].

The developing of strategies for taking advantage of simpler, cheaper and safer technologies, as well as resources and raw materials found in less-developed regions, makes the treatment of wounds more accessible to a large number of people. In this context, the research of natural products in the development of compounds to improve the healing process has been intensified in the last years [3]. A lot of plants, based on ethnopharmacological studies and its identified compounds, have been investigated for the potential use as wound healing agents, highlighting plants rich in antioxidant compounds and with anti-inflammatory activity [4–6].

The species *Curatella americana* Linn. is a member of the Dilleniaceae family, popularly known in Brazil as “lixeira or cajueiro-bravo”. This species is a woody perennial shrub that is characteristic of Neotropical Savanna, occurring from southern Mexico to Bolivia and in almost all savanna region of Brazil [7–9]. In the Amazonian savanna, *C*. *americana* is one of the most frequent species found in Amapá, Amazonas, Pará and Roraima states [10, 11].

The use of this plant as wound healing agent was described by Bailon (1871) in the book “The Natural History of Plants.” The author reported the use of stem bark to prepare an astringent lotion and the decoction of the leaves for topical application to wounds. In Brazilian folk medicine this species is used for inflammation, arthritis, bronchitis, high blood pressure [12], the leaf decoction is used as an antiseptic and astringent [13]; bark infusion is used for the treatment of cold, wounds healing and ulcers [14]. In Costa Rica, the cooked leaves are used to mitigate skin eruptions, for healing wounds, and the water for purifying blood [15, 16].

The leaves and bark extracts of *C*. *americana* have been described in the literature as anti-inflammatory, analgesic, antihypertensive, vasodilator, anti-ulcerogenic, antimicrobial and hypolipidemic agents [17–21]. Although, popularly used as wound healing agent, this activity was not validated.

The purpose of this study was to evaluate the wound healing potential of hydroethanolic extract and gel formulation from the leaves of *C*. *americana* through excision mouse wound model.

## Experimental section

### Collection and extraction of plant material

*Curatella americana* leaves were collected in the city of Macapá, State of Amapá, Brazil, in February 2015. A sampling location (00°9′ 75.251″ S, 51°8′ 57.6733″ W) was marked by a global position measuring (GPS Garmin nüvi 40). The scientific identification of the vegetable material was performed by a specialist and a voucher was deposited in the Herbarium of the Federal University of Amapá under the registration number 010266 for future reference.

Dried and powdered leaves were extracted with 70:30 (v/v) of ethanol:water. The crude extract was filtered in vacuum using Whattman^®^ filter. The hydroalcoholic extract was evaporated under vacuum using a rotatory evaporator (IKA® RV 05 basic), lyophilized and kept at –20°C in a freezer until further use and to be incorporated into pharmaceutical formulations.

### Animals

Adult Swiss albino mice (30–40 g) of either sex was used in all experiments. The animals were kept in polypropylene cages with access to water and food *ad libitum* under controlled temperature (18–20°C) on a 12 h light/dark cycle. All the experiments procedure and protocols involving animals were approved by the Ethics Committee of the Federal University of Amapá, under the register number 0015/2015.

### Microbiologic control of plant extract

The microbiologic control was made before the analyses to ensure the quality of the results. The tests for identification of colony forming unities (CFU) were performed for bacterial and fungi. The bacterial pathogen identification (*Escherichia coli*, *Salmonella sp*, *Pseudomonas aeruginosa*, *and Staphylococcus aureus*) was performed in the presence of CFU, according to Brazilian Pharmacopoeia [22]. The number of viable pathogens obtained (CFU/g) from the sample was compared with Guidelines for Assessing Quality of Herbal Medicines regarding Contaminants and Residues [23].

### The minimum inhibitory concentration (MIC) and the minimum bactericidal concentration (MBC)

Antibacterial activity was performed in three replicates and evaluated on the following standard strains: (i) Gram-positive bacteria: *Staphylococcus aureus* (ATCC 6538), *Staphylococcus epidermidis* (ATCC 12228) and (ii) Gram-negative bacteria: *Escherichia coli* (ATCC 8739), *Klebsiella pneumoniae* (ATCC 4352), *Pseudomonas aeruginosa* (ATCC 25853).

The HECA extract was dissolved in a solution of water and dimethylsulfoxide (DMSO) for the MIC and MBC assays. The analysis was performed using the broth microdilution method in the Mueller–Hinton broth as described by the Clinical and Laboratory Standards Institute [24]. The 3-(4,5-dimethylthiazol-2yl)-2,5-diphenytetrazolium bromide (MTT) was used as a bacterial viability indicator, because it is reduced by metabolically active cells to a colorful water-soluble formazan derivative [25]. The MIC was read as the lowest concentration of the extracts at which a change in color occurred. To the determination of the MBC, 10μL of broth was taken from each well and incubated in Mueller–Hinton agar at 37°C for 24h. The MBC was defined as the lowest concentration of extracts or fractions that resulted in either no growth or fewer than three colonies (99.9% killing) as described by Lopes et al. [26]. The negative control consisted of the bacterial inoculum in 100μL of DMSO 0.4% solution and as positive control trimethoprim associated with sulfamethoxazole.

### Coagulation assays

The fibrinogen coagulation (FC) assay was performed according to the methodology previously published by Moura and coworkers [27]. Briefly, the extracts solutions were incubated for 10 min at 37°C with human fibrinogen (Sigma Aldrich^®^) solution (2 mg/mL) in a final content of 250 μL then thrombin (200μg/mL) was added and the coagulation time was monitored. Prothrombin time (PT) assays were performed according to the manufacturer’s instructions (Wiener laboratories®). The extract was incubated with a commercial control plasm (Wama diagnostic ®) during 10 min at 37° C, and then, 100 μL of pre-warmed thromboplastin with calcium were added to initiate coagulation. The HECA was tested in different concentrations (500, 250, 200, 100, 50, 20 and 10 μg/mL) and the experiments controls were performed using water with plasma or fibrinogen solution. Coagulation time was recorded in seconds, and the assays were performed on a Multichannel Coagulometer (CloTimer).

### Folin-Ciocalteau assay

The Folin-Ciocalteau colorimetric method is based on the transfer of electrons in alkaline medium from phenolic compounds to phosphomolybdic/phosphotungstic acid complexes to form blue complexes [28]. Measurements were carried out according to the methodology previously published by reaction of the Folin-Ciocalteau reagent 10% (v/v) with Na_2_CO_3_ 700mM and samples diluted in water, for 2 hours and measured spectrophotometry visible at 760 nm using Biospectro SP-22. The assay was developed in triplicate and calculations based on a calibration curve obtained with gallic acid. The results were expressed as milligram of gallic acid equivalents (GAE) per g of dry extract [29].

### Liquid chromatography analysis coupled with high resolution mass spectrometry

LC-MS analysis was performed on a XEVO G2-STof Mass Spectrometer (Waters Corp., Milford, MA, USA). The acquisition parameters and chromatographic analysis were described in details previously, with some adjustments, for example, at the time of gradient elution to 20 minutes instead of 10 [29].

### Preparation of topical pharmaceutical formulation

Before the experiments, two types of formulations, aqueous solution and aqueous gel, with different concentrations 0.5 and 1.0% (w/v) of HECA were used in this study. The formulation is described in the National Formulary of Brazilian Pharmacopoeia, as follows: Polyacrylamide, C13-14 Isoparaffin and Laureth-7 (SEPIGEL^®^305) 4% (p/v); parabens solution 3,3% (v/v); 0.6% (v/v) of imidazolidinyl urea solution 50% and distilled water q.s.p. 100% [30].

All ingredients of the gel base were mixed until the required consistency, and then the lyophilized HECA were incorporated gently to obtain a uniform cream gel. Each 100 g of the gel prepared in the present study contained 0.5g or 1.0g of *Curatella americana* extract, called CaG 0.5% and CaG 1.0%, respectively. It was also prepared a gel without extract for control use, CV.

### Formulation stability

The samples of gel with HECA and the gel control were submitted to the physical preliminary and accelerate stability study described in the Cosmetic Products Stability Guide [31]. In the assays of preliminary stability were evaluated under centrifugations and the ice-thaw cycle for changes as volume loss, homogeneity, creaming and phase separation. In the accelerate stability, the pH measurements, organoleptic test, and the microbiologic assay were carried out after 24 hours, 30, 60 and 90 days with samples storage at ambient temperature (25°C ± 2); refrigerator (5°C ± 2) and drying oven (40°C ± 2).

After the end of the stability test, the microbiological quality of the samples was carried out to investigate the presence of UFC above the recommended limits and pathogen bacterial as *E*. *coli*, *Salmonella* spp., *P*. *aeruginosa*, and *S*. *aureus* as described previously for the extracts [22,23].

### Acute dermal toxicity

The OECD guidelines (N ° 402, 2017) [32], were followed for the study. A total of six animals (3 female and 3 male) mice were used. The limit test dose of 2000 mg/Kg of the 10% formulation was applied uniformly over the shaved area in a corresponding to 10% of body surface area. The shaved region was covered with sterile gauze attached to the ends with tape to prevent the animal from withdrawing or ingesting the product for 24 h. After the exposure period, the test substance was removed, and the clinical signs and mortality were monitored at 30 minutes, 2, 4, 6 and 24 hours and once a day until day 14. Individual body weights were measured just before administration (day 0), and on day 7 and 14 besides the volume of wather and food was measured every day.

### Groups and experimental protocols

The animals were randomly divided into six groups of ten animals each (a total of 60 mice). Each group of animals was treated with local application of: I) ointment containing bovine fibrinolysin 1U (Loomis), desoxyribonuclease 666 U and chloramphenicol 10 mg; (Fibrinase SA^®^; Pfizer S.A.) as positive control (FIB); II) gel without extract as control vehicle (CV); III) solution containing lyophilized extract of *C*. *americana* 0.5% (CaE 0.5%); IV) solution containing lyophilized extract of *C*. *americana* 1% (CaE 1%); V) gel containing lyophilized extract of *C*. *americana* 0.5% (CaG 0.5%); VI) gel containing lyophilized extract of *C*. *americana* 1% (CaG 1%). The treatments 100 mg or 100 μL/day were applied topically twice a day.

### Excisional wound model

Each mouse was weighed and anesthetized with the mixture of ketamine (90 mg/kg) and xylazine (10 mg/kg) injected intraperitoneally. After the loss of reflexes of the animals, trichotomy of the back was performed and antisepsis with 2% chlorhexidine gluconate. One cutaneous (full thickness, completely transdermal) circular wound of 6 mm diameter was made on the pre-shaved, dorsal surface of the animal with the help of a biopsy punch (Colgran 048-M). Animals were allowed to recover and were housed individually in polypropylene cages containing autoclaved paper cuttings. They received food and water *ad libitum*. Except for the drugs under study, no local/systemic therapy was provided to animals.

### Rate of wound contraction

One of the wounds was chosen for measure daily with pachymeter (ZAAS Precision-Digital 0–150 mm). The areas were calculated, starting from borders of wounds, using the equation A = π. R. r, where A represents the area (mm^2^), “R” the larger radius, and “r” the smaller radius. The calculation of the contraction degree was expressed in percentage using the follow equations [33].

$$\%\text{Wound contraction}=\frac{\left(\text{Initial wound size}–\text{Specific day wound size}\right)}{\text{Initial wound size}}\text{X}100$$

### Histopathology

On the 7th and 14th day of the treatment, five animals from each group were euthanized in a CO_2_ chamber for the collection of material for the morphometric analysis of the skin lesions. All the samples obtained were fixed in 10% buffered formalin for a minimum period of 24 hours and subsequently dehydrated in ethanol. Next, the samples were processed for inclusion in paraffin by routine methods and cut using a microtome set to 2.5 μm. The material obtained was placed on slides and coated with haematoxylin and eosin.

### Statistical analysis

The results are presented as the mean ± S.E.M. Differences among groups were analysed by one-way analysis of variance (ANOVA) followed by Tukey-Kramer test. The data were considered significant at p < 0.05 as compared to control group.

## Results

### Extract microbiologic analyse

The microbiologic analysis of HECA was performed to ensure the safety of the animals during all experiments. For dry extracts, a limited presence of microbial load is allowed, with a maximum of 10^4^ CFU / mL for bacteria and 10^3^ CFU / mL for fungi and yeasts and the absence of pathogenic bacteria [22]. The HECA was negative for bacteria and fungi contamination demonstrating the quality of the operational procedures in the extraction.

### Antimicrobial activity

Antibacterial screening revealed that the *C*. *americana* extract did not exhibit significant inhibitory effects as shown in Table 1.

**Table 1:** Minimum inhibitory concentration (MIC) and minimum bactericidal concentration (MBC) for the extract of *Curatella americana* Linn.

Bacteria	MIC (mg. mL^-1^)	MBC (mg. mL^-1^)
*Staphylococcus aureus*	>2.5	> 2.5
*Staphylococcus epidermidis*	2.5	2.5
*Escherichia coli*	0.62	>2.5
*Klebsiella pneumoniae*	>2.5	>2.5
*Pseudomonas aeruginosa*	>2.5	>2.5

MIC: minimum concentration inhibitory; MBC: minimum bacteriostatic concentration.

### Coagulation assays

The prothrombin time test measures how long blood takes to clot and the fibrin coagulation test evaluated the interference of the extract on fibrin clot formation. The extract did not exert any effect in the tested concentration over the fibrinogen neither in the prothrombin time.

### Folin-Ciocalteau assay and identification of compounds in hydroethanolic extract of *C. americana* leaves

The concentration of total phenolic compounds in the extract expressed as Gallic acid equivalent was 209 ± 4mg GAE/g of HECA.

**Fig 1:**
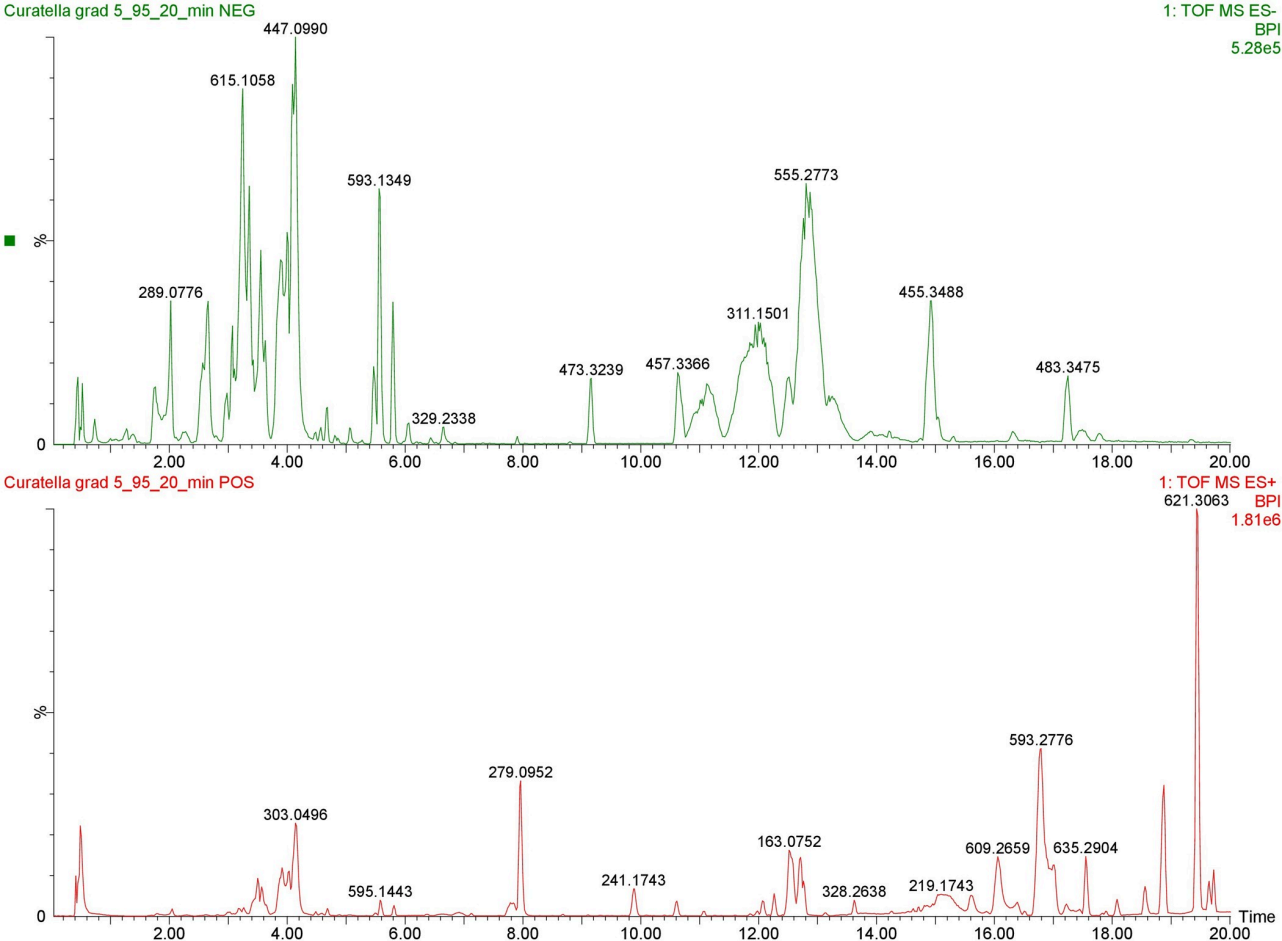
Total ion current chromatogram of hydroethanolic extract of *C*. *americana*.

The HECA extract was eluted by gradient mode and the total ion chromatogram (TIC) was acquired by UPLC-HRMS, on positive and negative mode. Nine compounds were identified at positive mode and four at negative mode. The identification of compounds was carried out by comparing compounds m/z ratios with those of metabolome platforms, considering an error ≤10 ppm, see Fig 1 and Table 2.

**Table 2:** Identified compounds in the HECA with their respective retention times and m/z ratio.

Number	Components	Empirical formula	Chemical class	RT (min.)	MS mode	Calcd.	Obsd.	Error
1	Catechin	C_15_H_14_O_6_	Flavan-3-ol	1.73	[M-H]^-^	289.0712	289.0706	2.1
2	Epicatechin	C_15_H_14_O_6_	Flavan-3-ol	2.03	[M-H]^-^	289.0712	289.0741	10.0
3	kaempferol-4-β-glucopyranoside	C_21_H_20_O_11_	Flavonol	2.73	[M+H]^+^	449.1084	449.1064	4.5
4	Quercetin-3- β-glucopyranoside	C_21_H_20_O_12_	Flavonol	3.48	[M+H]^+^	465.1033	465.1029	0.9
5	Apigenin-7-O- β-glucopyranoside	C_21_H_20_O_10_	Flavone	3.50	[M+H]^+^	433.1135	433.1136	0.2
6	Quercetin-3-O-galactopyranoside	C_21_H_20_O_12_	Flavonol	3.62	[M+H]^+^	465.1033	465.1029	0.9
7	Quercetin-3-O-arabinopyranoside	C_20_H_18_O_11_	Flavonol	4.01	[M+H]^+^	435.0927	435.0920	1.6
8	Quercetin-3-α-rhamnopyranoside	C_21_H_20_O_11_	Flavonol	4.14	[M+H]^+^	449.1084	449.1064	4.5
9	Quercetin	C_15_H_10_O_7_	Flavonol	5.51	[M+H]^+^	303.0505	303.0531	8.6
10	kaempferol-3-O-(6”-O-E-p-coumaroyl)-β-D-glucopyranoside	C_30_H_26_O_13_	Flavonol	5.54	[M+H]^+^	595.1452	595.1492	6.7
11	kaempferol-3-O-(2”-O-E-p-coumaroyl)-β-D-glucopyranoside	C_30_H_26_O_13_	Flavonol	5.77	[M+H]^+^	595.1452	595.1492	6.7
12	Kaempferol	C_15_H_10_O_6_	Flavonol	6.49	[M-H]^-^	285.0399	285.0374	8.8
13	Betulinic acid	C_30_H_48_O_3_	Terpenoid	15.00	[M-H]^-^	455.3525	455.3487	8.3

### Topical formulation and stability

The gel was chosen because it has been used the dermatological basis for all skin types besides being a polymer of low cost and easy manipulation, it presents smooth spreading, low occlusion and can be used for incorporation of several active ingredients, including antimicrobial and anti-inflammatory agents [34, 35].

The tests were conducted in triplicate and the samples stored under conditions that accelerate possible changes that can occur during the expiration date and show indications as to their stability. In the preliminary stability, any changes in volume, homogeneity, creaming and phase separation were observed.

The pH, organoleptic and microbiologic changes observed in the accelerated stability are shown in Table 3.

**Table 3:** Results of accelerate stability of gel formulations.

Conditions	pH			Color/Smell			Microbiological control		
	Gel 0.5%	Gel 1.0%	Gel control	Gel 0.5%	Gel 1.0%	Gel control	Gel 0.5%	Gel 1.0%	Gel control
Formulation day	5.15	5.08	7.01	I/I	I/I	I/I	NR	NR	NR
Ambient temperature (25°C ± 2)									
30 days	5.95	5.97	6.97	I/I	I/I	I/I	-	-	-
60 days	5.55	5.59	7.20	II/II	II/II	I/I	-	-	-
90 days	5.50	5.6	7.22	II/II	II/II	I/I	+	+	-
Refrigerator (5°C ± 2)									
30 days	5.98	5.97	6.98	I/I	I/I	I/I	-	-	-
60 days	5.08	5.03	7.20	I/I	I/I	I/I	-	-	-
90 days	5.07	5.01	7.01	II/I	II/I	I/I	-	-	-
Dry over (40°C ± 2)									
30 days	5.92	5.95	6.95	I/I	I/I	I/I	-	-	-
60 days	5.06	5.21	7.30	III/II	III/II	I/I	-	-	-
90 days	4.07	4.48	6.64	III/II	III/II	I/I	-	-	-

NR: not realized; (I) Normal, (II) slight modified, (III) modified, (III) intensely modified. **+**: means <10^1^ UFC/mL, for fungi.

For non-sterile topical products, a limited presence of microbial load is allowed, with a maximum of 10^2^ CFU / mL for bacteria and 10^1^ CFU / mL for fungi and yeasts and the absence of pathogenic bacteria [22]. In the analyzed formulations there was no growth of microorganisms above the recommended and no presence of pathogenic bacteria.

### Acute dermal toxicity

The limit dose tested was found to be safe (2000 mg/Kg of the 10% formulation). In this test, no erythema, eschar, edema, symptoms of toxicity, mortality or any other reactions were observed in either animals and we classified the *C*. *americana* as 'non-irritant', at the tested concentrations.

### Wound contraction

There were no differences in the wound contraction between all formulations with the hydroalcoholic extract of *C*.*americana* and the positive control (Fibrinase®, FIB), see Table 4. The group *C*. *americana* extract (CaE) 1% presented the best rate of wound contraction in the seventh day.

**Table 4:** Effect of topical application of the formulation and aqueous solution from hydroalcoholic extract of *Curatella americana* L.

Group	0 day	7^th^day		14^th^day	
	Wound area (mm^2^)	Wound area (mm^2^)	% wound contraction	Wound area (mm^2^)	% wound contraction
CV	29.6 ± 4.05	11.46 ± 4.79	61	0.58± 1.29	98
FIB	25.16 ± 2.75	5.79 ± 3.6*	78	0.35± 0.79	99
CaE 0.5%	27.14 ± 4.44	7.36 ± 3.54	73	0	100
CaE 1%	30.02 ± 4.32	4.51 ± 1.15**	85	0	100
CaG 0.5%	26.45 ± 6.7	6.69 ± 1.85*	74	0	100
CaG 1%	28.02 ± 3.16	6.88 ± 2.37*	75	0.16 ± 0.36	99

Values are expressed as mean ± SEM and analyzed by one-way ANOVA followed by the tuckey- Kramer. In the 0 and 7^th^ day, 10 animals were considered.

* *p*< 0,05;

**p< 0,01, against vehicle control (CV). CaG: *C*. *americana* gel, CaE: solution of *C*. *americana* extract.

### Histopathological examination

**Fig 2:**
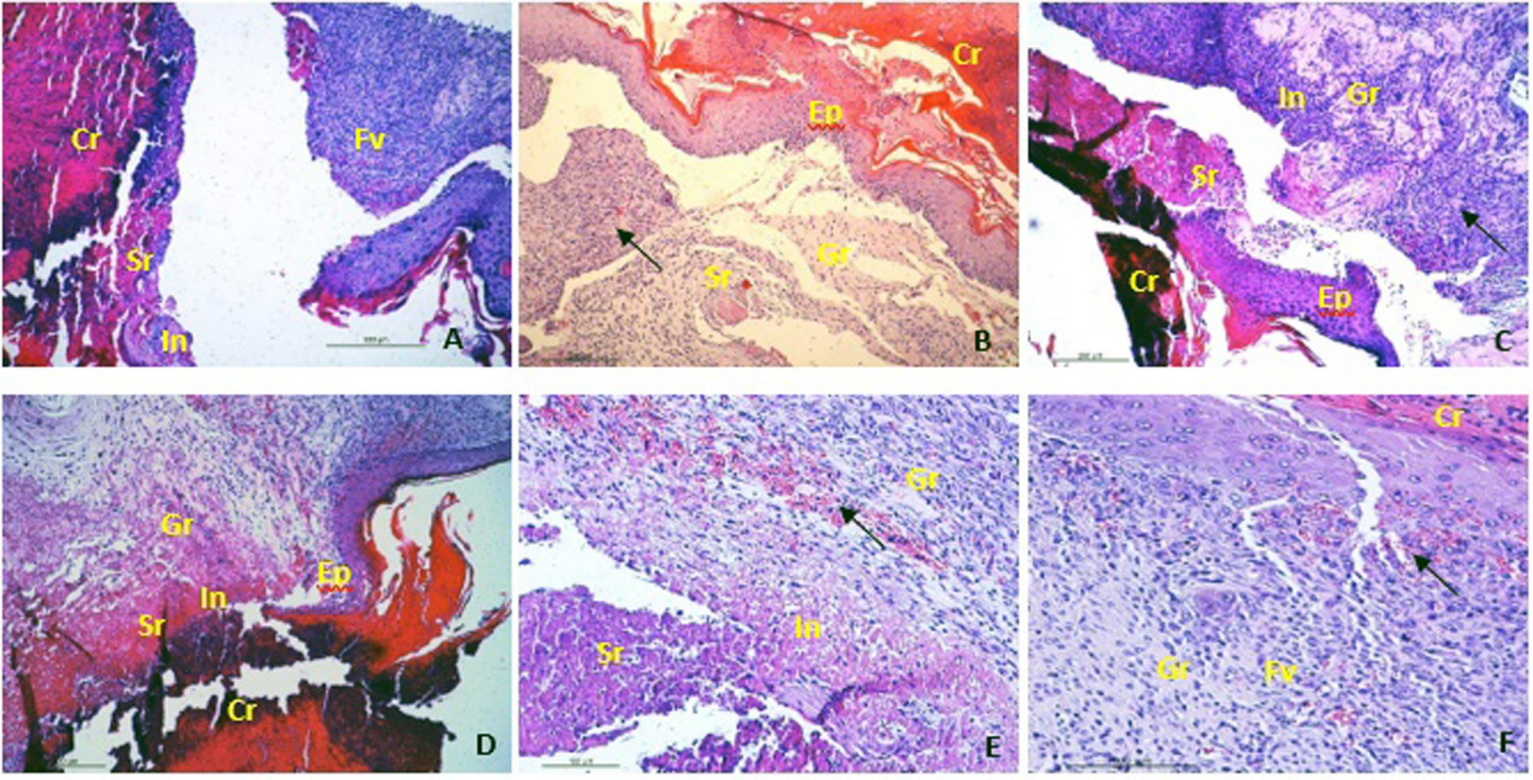
Sections stained with haematoxylin-eosin (x40) on 7 ^th^ day. (A) group CV; (B) group FIB; (C) group CaG 0.5%; (D) group CaG 1%; (E) group CaE 0.5% and (F) group CaE 1%. Cr: crust with piocytes, FB: fibroblasts, N: neovasal, Sr: seroma, Ep: epithelization, In: Inflammatory cells, Fv: fibrovascular proliferation, Black arrow indicating granular tissue with initial remodeling.

Histopathological analysis of the seventh day showed that all groups treated with *C*. *americana* and FIB group had epithelialization, fibroplasia, and vascularization active. It was possible to observe the tissue in initial remodelling and stages of recent granulation in a lower proportion in the CaG 1% group. In the CaG 0.5%, CaG 1% and CaE 0.5% groups, the presence of inflammatory cells such as neutrophils and macrophages were observed. In the CV group, the cicatricial process was slowed by the presence of extensive seroma with neutrophil infiltration despite the presence of bulky fibroblasts and angiogenesis, see Fig 2.

**Fig 3:**
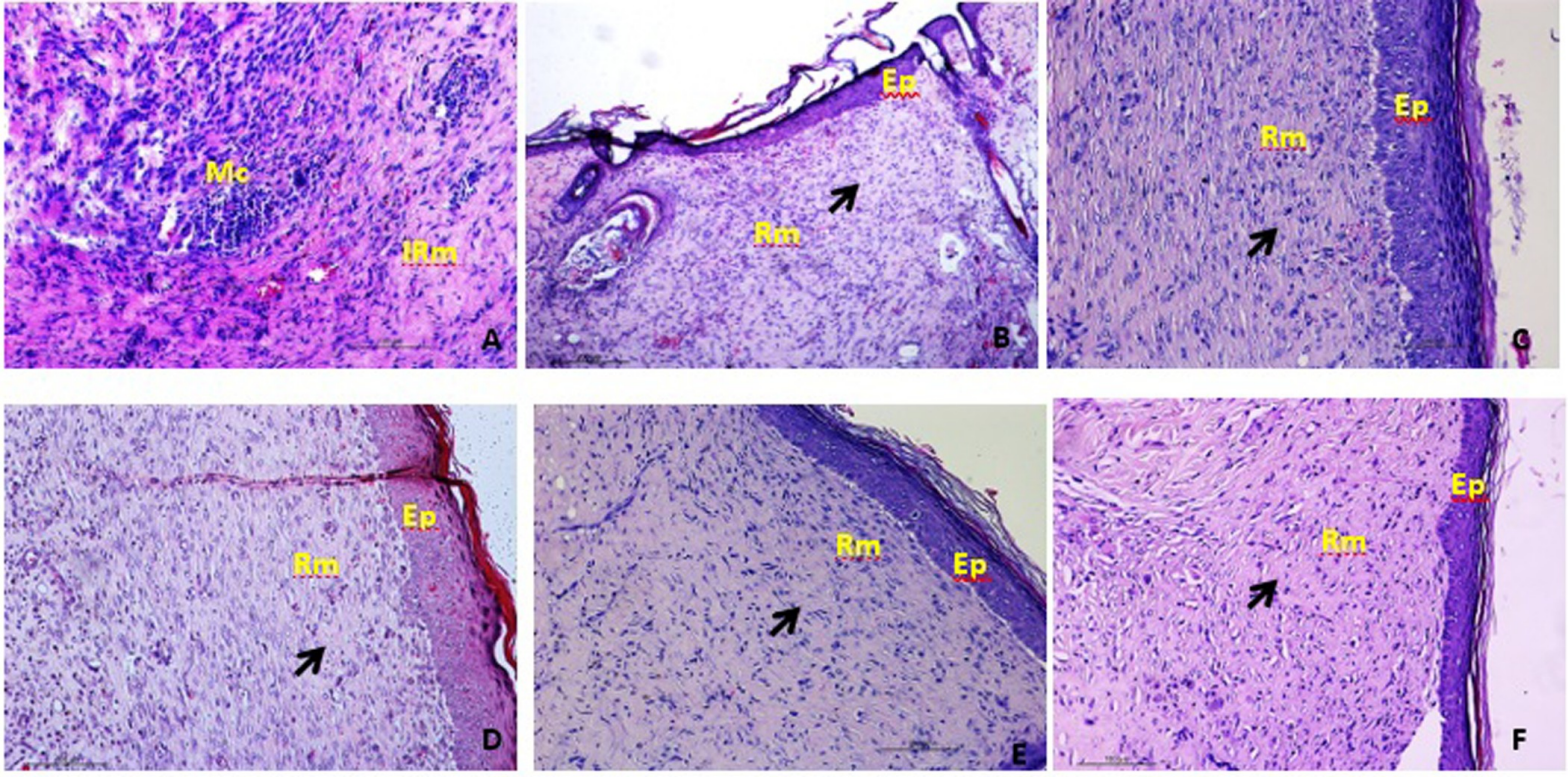
Sections stained with haematoxylin-eosin (x40) on 14 ^th^ day. (A) group CV; (B) group FIB; (C) group CaG 0,5%; (D) group CaG 1%; (E) group CaE 0.5% and (F) group CaE 1%. Ep: epithelization, Mc: Microabcesses, IRm: initial remodelling, Rm: advanced remodelling, Black arrow indicating collagenization.

On the 14th day, it was possible to observe in all groups treated with HECA complete epithelization and tissue remodelling at an advanced stage with denser collagen fibers like the FIB group. While in the CV group, despite re-epithelialization and early stages of remodeling, impairment was observed in the cicatricial process due to the presence of microabscesses (Fig 3).

## Discussion

The wound healing is a complex physiologic process that involves blood clothing, inflammatory response, re-epithelization, and angiogenesis. These processes are divided into three phases: inflammatory in 0–3 days, cellular proliferation between 2–14 days and remodelling initiated in 14 days and may persists months [36,37]. One of the leading causes of delayed healing is the persistence of inflammation, mainly for reactive oxygen species (ROS), infectious processes or an inadequate angiogenic response [38].

Based on this, an excellent healing agent should combine the antimicrobial activity, antioxidant activity and mainly having enhanced mitogenic activity to increase cell proliferation, and consequently angiogenesis making possible the faster contraction.

The time that the blood takes to clot is clinically relevant for the homeostasis in the skin injury [39]. Although any concentration test of HECA presented coagulant effects, this did not inhibit the normal process of coagulation. After clot formation, the cellular response is established 24 hours after injury, and neutrophils initiate debridement of the devitalized tissue and phagocytosis of infectious agents through the release of lysosomal enzymes and reactive oxygen species (ROS). In 48 hours the macrophages migrate to the site of the lesion contributing to the end of the cleaning and disinfection initiated by neutrophils and producing ROS [36,37].

Inappropriate scavenging of these ROS generally results in a delay of wound healing, individuals with chronic leg ulcers and lesions due to ischemia present excessive production of oxygen radicals with significant xanthine oxidase activity [40].

In this context, plant extracts with healing activities have their effect justified by the presence of metabolites capable of capturing oxygen radicals [3,41,42]. The HECA extract showed an important concentration of phenolic compounds (209 mg / g GAE) confirming the potent antioxidant activity of this species through the capture of ROS previously demonstrated by Lopes et al. and Fujishima et al. [21, 43].

The mass spectrum of HECA revealed the presence of flavonoids, terpenoids, phenolic derivatives confirming studies performed by El-Azizi et al. and Gurni & Kubitzki [44,45]. Other wise, in this study was possible identify catechin and epicatechin by comparison with injection of authentic standards. These compounds have been previously found in the bark [18, 20], besides kaempferol and apigenin- 7-O- glucoside that were never identified in this specie before.

Among the identified phenolics, flavan- 3- ols as catechins and epicatechin has shown significant antioxidant activity including the potential inhibitory effect on xanthine oxidase [46]. Besides, the pretreatment of fibroblasts cells with catechin accelerated the proliferative response even after induction of oxidative stress [47].

Hiruma-Lima and coworkers, identified in *C*. *americana* bark extract monomeric and polimeric catechins beyond of mixture of procyanidins, that could explain the excellent healing activity on gastric ulcers in addition to gastroprotective effect by increasing the production of prostaglandin E2 (PGE2) in the mucosa [20] and consequent induction of significant increase in vascular endothelial growth factor (VEGF) expression leading to angiogenesis [48,49].

The critical role of PGE2 in the healing process and fibroblast proliferation is confirmed by the fact that immediately after the injury the cyclooxygenase 2, (COX 2), the primary enzyme producing PGE2, is induced in the epidermis and the assays in which the use of specific inhibitors of COX-2 led to delays in healing [50]. The intake of catechins from the green tea for 12 weeks induced the incorporation of catechin metabolites into human skin associated with abrogated UVR-induced pro-inflammatory mediators 12-hydroxyeicosatetraenoic acid (12-HETE) but did not reduce PEG2 [51].

Plant extracts rich phenolics compounds including catechin and epicatechin stimulated the growth of ephitelial and fibroblast cell as well as enhance the vessels formation *in vitro* [52, 53], catechin was able to increase the viability of human umbilical vein endothelial cells (HUVEC), reducing apoptosis, but it contoled the angiogenesis in excess of VEGF [54].

The compounds kaempferol and apigenin- 7- O- glucoside have never been identified before in this specie. Regarding quercetin and its derivatives, anti-oxidant and anti-inflammatory activities have been attributed in the same way the kaempferol, apigenin and its derivatives [55,56]. While apigenin and kaempferol strongly inhibit COX-2 induction by inhibiting factor-kB nuclear transcription factor (NF-kB) via kB-inhibitor, on the other hand, the derivatives of these compounds as well as quercetin and catechins appear to slightly inhibit COX- 2 [55], this is important to modulate the inflammation. Topical application of quercetin appears to be effective in treating wounds by enhancing granular tissue formation, enhancing fibroblast proliferation, and collagen production with decreased inflammatory cytokines, including in diabetic animals [57,58].

The betulinic acid, single terpenoid identified, appear inhibiting the production of metalloproteinases (MMPs) [59]. The action on metalloproteinases such as collagenases and elastases, is important for healing, because the excessive production of MMPs leads to delayed healing by the absence of a matrix for the anchorage of the new cells which may result in a chronic lesion [60, 61, 60].

Based on the identified compounds, it is possible to suggest that the stimulatory action on fibroblasts and endothelial cells as well as inflammatory modulation contributed with the high rates of wound contraction (86% for the CaE 1.0% group) in the groups treated with *C*. *americana*. The percentage of contraction on the seventh day differed significantly from the CV group, however, with similar indexes to the positive control group (FIB). High contraction rates on the eighth day were also identified with the extract of the stem bark of *Acacia leucopholea*, *Embelia ribes* and *Rumex abyssinicus* [33,62,63].

The fibrinase is composed of lytic enzymes, fibrinolysin and deoxyribonuclease, in the form of a mild emollient ointment containing 1% of chloramphenicol and is indicated for the treatment of infected lesions such as burns, ulcers and second intention wounds, where the double action as debriding agent and topical antibiotic is required [1]. The evaluation of the antimicrobial activity of the HECA confirmed low activity of this plant species [18,64]. Despite this, HECA proved to be efficient in the healing process, evidenced by histopathological analysis.

In the proliferative phase, 2 or 3 days after the injury, the fibroblasts appear and dominate the cellular population until the first week. First its activity is confined to cellular replication and proliferation followed by the collagen synthesis that characterizes fibroplasia. Angiogenesis accompanies the fibroblast phase and is essential for healing since the new vessels will provide nutrients and oxygen for the formation of granulation tissue. If angiogenesis is inefficient, the fibroblast migration is slow, and the healing process fails. In this stage also begins the epithelization of the wound with the migration of the epithelial cells from the border toward the center [35,36].

On the seventh day of the experiment, the histopathological analysis showed that all groups treated with *C*. *americana* and FIB group had an area with epithelialization, fibroplasia, and vascularization active with fresh granulation tissue. It is possible to observe in these groups tissue in initial remodeling demonstrating stage of advanced healing considering that the period of remodeling usually begins around the 12^th^ day [36].

At this stage, the CaE 1.0% group was more effective because it presented contraction values closer to fibrinase with the absence of inflammatory cells in the histopathological analysis. It is important to emphasize that the choice of extract concentrations was based on previous analyzes in which the 0.4% extract presented good healing activity [65], however, still with inflammatory processes that were observed in the CaE 0.5% group but did not affect the healing quality in the phase of remodeling, day 14, where all the groups, except CV group, presented excellent cicatricial process.

The gel carrier also appears to have influenced this phase since the *C*. *americana* based gel treated groups exhibited inflammatory cells, even at 1% concentration, suggesting that the debridement process would still be occurring. Kant and coworkers [66], in a study with water-based polymeric gel, showed that this vehicle increased cutaneous healing in rats compared to saline, evidencing an increase in the number of leukocytes in the inflammatory phase, but with better contraction rates and cicatricial quality than the control.

As the polymer used in this study is considered inert and non-irritating up to 5% concentration [67], this event can be explained by the moisture generated by these vehicles. Because the wet environment of the wound accelerates healing by preventing cellular dehydration increasing its viability, stimulating collagen synthesis and angiogenesis [68, 69]. It is possible to suggest, therefore, that the aqueous gel vehicle together with the proliferative effects of quercetin and maintenance of cell viability of catechin would contribute to this process [70]. Alternatively, the chosen vehicle has merely impaired the release of anti-inflammatory actives from the extract. The influence of the gel vehicle on the cicatricial process was also observed by Barros and co-workers, where the extract presented a percentage of healing superior to the gel formulation in cutaneous wound models in rats [71].

The formulation tested was shown to be safe for the incorporation of *C*. *americana* extract and with significant healing activity. All organoleptic changes in formulations stored at room temperature and refrigerated were acceptable according Brazilian legislation [31]. On the other hand, intense modifications occurring at 40°C are considered as decomposition of the formulations due to oxidative processes and loss of volatile compounds, therefore, the formulation is considered to be disapproved [31].

There was a decrease in pH, remaining around 4.4 ~ 5.9, probably due to chemical constituents such as flavonoids, but within the physiological pH of the skin that is slightly acidic (4.6 ~ 5.8), thus avoiding undesirable reactions of the skin to the product, such as irritation, redness, pruritus and others, besides contributing to the protection of bacteria and fungi on its surface [72].

The data generated from the present study indicated that the *Curatella americana* extract increased the wound contraction and accelerated the wound healing, probably by modular the immune response. These findings confirming the traditional use of these specie in wound healing. However, the choice of the gel vehicle for wound treatment should consider the characteristics of the lesions to be treated and the clinical conditions of the patients, suggesting their use only in dry lesions.
